# Surgical resection of lumbar intradural metastatic renal cell carcinoma

**DOI:** 10.3171/2023.7.FOCVID2379

**Published:** 2023-10-01

**Authors:** Mohamed Macki, Vardhaan S. Ambati, Christine Park, Michael Tawil, Abraham Dada, Alysha Jamieson, Sean Wilkinson, Timothy Chryssikos, Praveen V. Mummaneni

**Affiliations:** Department of Neurological Surgery, University of California, San Francisco, California

**Keywords:** cauda equina tumor, intradural tumor, metastatic renal cell carcinoma, intraoperative ultrasound, neuromonitoring checklist, spinal metastasis, motor evoked potentials

## Abstract

A 60-year-old male with renal cell carcinoma (RCC) presented with back pain, weakness, and bowel and bladder urgency. MRI demonstrated a cauda equina tumor at L2. Following L1–3 laminectomies, intraoperative ultrasound localized the tumor. After dural opening, a vascular tumor was adherent to the cauda equina. Intraoperative nerve stimulation helped to identify the nerve rootlets. Tumor was removed in a piecemeal fashion. Tumor dissection caused periodic spasms in L1–3 distributions. A neuromonitoring checklist was used to recover motor evoked potential signals with elevated mean arterial pressures. Hemostasis was challenging with the vascular tumor. Intraoperative ultrasound confirmed tumor debulking. Pathology confirmed metastatic RCC.

## Transcript

We present the technical nuances for surgical resection of intradural, metastatic, renal cell carcinoma.^1^

### 0:25

Intradural metastasis of renal cell carcinoma is a rare event,^2^ with only 19 published cases according to a recent literature review.^3^ The survival rate was approximately 80% in the 15 patients with 25 months’ follow-up. Here, we describe one of the first surgical videos of a renal cell carcinoma that metastasized to the cauda equina. Although the prognosis may be poor, surgery should be considered in symptomatic patients with motor weakness and bladder dysfunction if they harbor a solitary, intradural spinal mass in the absence of leptomeningeal disease.

### 1:02

Sixty-year-old male with history of metastatic renal cell carcinoma presents with back pain, radiculopathy, and bowel and bladder urgency. Examination demonstrates weakness in multiple muscle groups in the bilateral lower extremities. Preoperative MRI included T2-weighted images and T1-weighted postcontrast images, which are consistent with an intradural tumor with hemorrhagic blood products and serous loculations. Tumor was within the cauda equina, centered at L2, just below the conus medullaris.

### 1:36

Oncological advancements have noted favorable response rates with immunomodulator therapies for renal cell carcinoma.^1^ While the current patient was already treated with checkpoint inhibitors for his metastatic disease, the multidisciplinary team felt that the solitary intradural tumor would respond well to adjuvant tyrosine kinase inhibitors and stereotactic body radiation therapy after surgical resection. In patients with bladder dysfunction, urgent surgery is especially important since renal cell carcinoma is resistant to external-beam radiation therapy.^1,4^

### 2:10

The operation included an L1–3 laminectomy for intradural resection of tumor. Neuromonitoring was used.^5,6^ After obtaining preflip baseline neuromonitoring signals, the patient was positioned prone on a spinal Jackson with the Wilson frame. L2 was localized with C-arm fluoroscopy.

### 2:28

An incision centered around L2 was completed with a No. 10 blade scalpel. Subperiostal dissection was completed in standard fashion. Laminectomy was completed with Horsley bone cutter, rongeurs, and high-speed matchstick burr. Meticulous hemostasis ensures epidural bleeding does not contaminate the field. Following irrigation of the surgical cavity, the ultrasound ensures that the tumor is within the laminectomy field.^7^ Both the axial and sagittal cuts of the ultrasound are shown here. Important landmarks of the ultrasound are also labeled. The initial durotomy ensures that the arachnoid plane is preserved. Next, a microporous monofilament suture of flexible biomaterial is used to tack up the dura.

### 3:33

The arachnoid plane is carefully dissected. After the dural and arachnoid opening, a vascular tumor was found to be adherent to the cauda equina. Intraoperative nerve stimulation identified the underlying nerve rootlets. Then, a plane was established to prepare for piecemeal dissection. The loculations of intratumoral hemorrhage and serous fluid were intentionally preserved. This maintained the tumor turgor, which permits for a more facile dissection and release from the adherent nerve roots. Ultra-thin cotton patties are placed in the established plane between the tumor and nerve roots. If the surgeon chooses to return to a certain region of the tumor, the tumor plane is maintained with these neurosurgical patties.

### 4:25

Once the patties circumferentially surround the tumor capsule, the final bulk of the pathological tissue can be removed. Any residual tumor is then removed until the ventral dura is appreciated. Direct stimulation aids in distinguishing the filum terminale from the nerve roots. This highlights the importance of neuromonitoring in these cases. During tumor dissection, EMG activity was consistent with spasming of the muscles innervated by the L1–3 nerve roots. This corresponded to a transient decrease in the motor evoked potentials, secondary to manipulation near the conus. In these situations, a neuromonitoring checklist was developed to guide the entire operating room team.^8,9^

### 5:17

In this case, neuromonitoring signals returned with pharmacological elevation of the mean arterial pressure. If the motor evoked potentials do not return, direct waves or D-waves may be considered. Although not needed here, we were prepared to place electrodes if the neuromonitoring signals did not respond. Once signals return, the surgery may proceed cautiously. Intraoperative pathology confirmed metastatic renal cell carcinoma. In light of this diagnosis, after sufficient tumor has been debulked, meticulous hemostasis is prudent given propensity of renal cell carcinoma to bleed. Hemostatic matrix mixed with thrombin can be injected into the dural cavity. After copious irrigations and meticulous bipolar cautery, hemostasis is achieved. The surgical field is thoroughly inspected for any large surgical residual tumor. Here, the ultrasound confirmed that the tumor was appropriately debulked.

### 6:32

Following ultrasonic verification, the tack-up sutures are cut, and the dura is closed with an interlocking suture. We prefer microporous monofilament suture of flexible biomaterial, which has the additional benefit of a needle that is smaller than the suture. This decreases the likelihood of a cerebral spinal fluid leak through the suture holes. Prior to the final stitching, the intradural space is filled with lactated Ringer’s solution. A fibrin sealant patch is then used instead of a fibrin sealant glue, which may cause artifactual enhancement on the postoperative MRI. The wound is closed in multiple layers in a watertight fashion. The patient tolerated the procedure without complication. He was discharged on postoperative day 4, and examination improved to full strength in the lower extremities. Postoperative MRI confirmed excellent tumor resection.

### 7:33

Six weeks after surgery, the patient was treated with adjuvant immunomodulators and radiation.^1,4^ The patient will continue to be followed with serial imaging. Importantly, however, given the paucity of cases, recurrence rates after surgical resection of intradural renal cell carcinoma metastasis has not been previously established. Thank you for your attention.

## Disclosures

Dr. Mummaneni reported personal fees from DePuy Synthes, Globus, NuVasive, Stryker, BK Medical, Brainlab, and SI Bone; book royalties from Thieme Publishing and Springer Publishing; grants from AO Spine, NIH/NIAMS (U19AR076737), PCORI, Pacira, NREF, and ISSG; and stock ownership in Spinicity/ISD, outside the submitted work.

## Author Contributions

Primary surgeon: Mummaneni. Assistant surgeon: Macki. Editing and drafting the video and abstract: Ambati, Macki, Tawil, Dada, Jamieson, Wilkinson, Mummaneni. Critically revising the work: Ambati, Macki, Dada, Chryssikos, Mummaneni. Reviewed submitted version of the work: Ambati, Macki, Park, Tawil, Dada, Jamieson, Chryssikos, Mummaneni. Approved the final version of the work on behalf of all authors: Ambati. Supervision: Macki, Chryssikos. Illustrations: Park.

## Supplemental Information

### Patient Informed Consent

The necessary patient informed consent was obtained in this study.
